# Genetic Diversity of Bovine Viral Diarrhea Virus Infection in Goats in Southwestern China

**DOI:** 10.1155/2018/8274397

**Published:** 2018-11-18

**Authors:** Yu Deng, Silu Wang, Runxia Liu, Guiying Hao

**Affiliations:** ^1^School of Animal Science, Xichang College, Xichang 615013, China; ^2^South Dakota State University, Brookings, SD 57007, USA

## Abstract

Bovine viral diarrhea virus (BVDV) affects cows, pigs, sheep, goats, and other ruminants, as well as some wild animals. BVDV causes considerable economic losses every year and many countries have developed programs aimed at the eradication of this disease. The genetic diversity of BVDV in diseased goats has never been described in southwestern China. Thus, in this study, we applied antigen-capture ELISA and RT-PCR to survey the infection rate of BVDV in diseased goats in this region. Our results demonstrated that the average BVDV infection rate in goats was 17.51%, with all positive samples indicating infection by BVDV-1 and not BVDV-2, BVDV-3, or Border disease virus. The molecular characteristics of the 5′-untranslated region (5′-UTR) of BVDV-1 were recognized as belonging predominantly to the BVDV-1a, 1b, 1c, 1m, and 1p subtypes. BVDV-1b and 1m were the most abundant subtypes identified in this region, similar to the BVDV epidemics in cattle in other regions of China. This is the first study that describes the genetic characterization of BVDV in sick goats from southwestern China and is important for future studies and control programs.

## 1. Introduction

Bovine viral diarrhea virus–1 (BVDV–1) and BVDV–2, together with classical swine fever virus and border disease virus (BDV), are currently classified as the genus* Pestivirus* and family* Flaviviridae* [1]. A new unclassified* Pestivirus* species, HoBi-like virus, was first identified in fetal bovine serum from Brazil and was related to BVDV at the antigenic and genetic levels. Thus, the viruses were referred to as “BVDV–3” [2]. To date, a growing number unassigned atypical* Pestivirus *species have been detected in Europe, North America, South America, and Asia [3]; however, such emerging pestiviruses have not been officially recognized. To resolve the taxonomy of pestiviruses, a new taxonomy for the genus* Pestivirus *(family* Flaviviridae*) was proposed, where all species were redesignated as* Pestivirus A-Pestivirus K* [4].

The natural host of BVDV is cattle though it is also capable of infecting sheep, goats, piglets, and other domestic and wild ruminants by persistently infected (PI) animals and transiently infected individuals [5]. PI cattle are the main source of BVDV; however, persistent infections also occur in nonbovid hosts such as sheep and deer. BVDV infections in goats typically result in reproductive diseases and viable PI goats are rare [6]. There is increasing evidence that BVDV infections occur in a wider range of species, including mountain goats and domestic goats that have been reservoirs for BVDV infection and seriously impact the domestic livestock industry.

BVDV has a single stranded positive sense RNA genome of 12.3–12.5 kb. The full-length viral genome is flanked at both ends by 5′ and 3′ untranslated regions (UTRs) and contains a single open reading frame that encodes a single polyprotein that is approximately 4000 amino acids. The genetic diversity of novel* Pestivirus* strains is usually based on the characteristics of partial sequences from the 5′–UTR, N^pro^ or E2 regions of the genome. However, the 5′–UTR of Pestiviruses has the highest degree of sequence conservation and is therefore most frequently used for phylogenetic analysis. According to sequence comparison analyses of this region, there are 21 subtypes of BVDV–1(1a–1u) [7–9] and four subtypes of BVDV–2 (2a–2d) [10–12].

A previous study based on a strain of BVDV–1 that infected goats in eastern China showed that a high proportion (29/236) of BVDV–positive goats came from eastern China [13]. However, studies regarding the genetic diversity of BVDV in diseased goats in southwestern China remain rare. Thus, this study detected and genotyped BVDV from infected goats in this region.

## 2. Materials and Methods

### 2.1. Samples

A total of 217 blood samples taken from diseased goats that presented with symptoms, such as diarrhea, respiratory tract infection, and mucositis, were collected between March 2013 and April 2016, from multiple goat farms in southwestern China.

### 2.2. BVDV Antigen-Capture ELISA (ACE)

Sera were screened for BVDV using IDEXX BVD Ag Test (IDEXX laboratories Inc., Shanghai, China), which can be applied for detection of genetically diverse BVDV strains, according to the manufacturer's instructions. The antigen-positive samples were then followed up with RT-PCR detection.

### 2.3. RNA Isolation and RT-PCR Detection of BVDV–1, BVDV–2, BVDV–3, and BDV

Viral RNA was extracted from 200 *μ*L aliquots of goat serum using the QIAamp Viral RNA Mini Kit (QIAGEN China, Shanghai, China), following the manufacturer's recommendations. Four different PCR assays for BVDV–1 [14], BVDV–2 [15], BVDV–3 [16], and BDV [17] were performed to amplify the respective targets. The PCR products were purified and sequenced by the JIELI Biology Company (Shanghai, China).

### 2.4. Phylogenetic Analysis

The 5′–UTR genomic sequences obtained were compared with the relevant sequences published in GenBank (http://www.ncbi.nlm.nih.gov/genbank) using the Basic Local Alignment Search Tool (BLAST). The sequences in this study were trimmed and analyzed with MEGA7.0 software [18] to obtain 224 nucleotide sequences, corresponding to the 5′–UTR region of BVDV. The 224 nucleotide sequences were then subjected to alignment using the ClustalW program using MegAlign in the Lasergene 12.0 software (DNASTAR Inc. Madison, WI, USA), and genetic diversity analysis was performed using MEGA7.0 software. The evolutionary distances in the phylogenetic tree were computed using the Tamura 3–parameter model with 1000 bootstrap replicates, each constructed by the Neighbor-Joining method [18].

## 3. Results

The results of ACE showed that out of the 217 diseased goat sera samples tested using ACE, 30 were antigen-positive for BVDV. Antigen-positive samples were confirmed as BVDV-1 using BVDV-1 specific nested RT-PCR. Fifteen additional samples that were close to the cutoff value of ACE were also tested with nested RT-PCR, eight of which were positive for BVDV-1. Taken together, 38 sequences from 217 total samples corresponded to BVDV–1 by nested RT-PCR, which resulted in a 197–bp amplicon from the BVDV–1 5′–UTR. However, BVDV–2, BVDV–3, and BDV were not detected in any of these samples using RT-PCR. These results indicated that the average infection rate of BVDV–1 in goats was 17.51% (38/217) in southwestern China.

BVDV–1 was detected in 12 of 38 BVDV–positive samples by first round RT-PCR, which produced a 305 bp product that was not detected in the other 26 BVDV–1–positive samples. The sequences detected with the first round RT-PCR in the 12 BVDV–1 positive samples were deposited in GenBank under accession numbers MG323516–MG323527 (Table 1).

Twelve of those sequences were selected for the construction of a phylogenetic tree and were divided into five different subtypes. Five isolates (XC1522, HL1407, DC1301, SC1601, and MN1319) were clustered within BVDV–1b, one (DS1324) was typed as BVDV–1a, one (FS1573) as BVDV–1c, one (FS1426) as BVDV–1p, and four (FS1605, CF1374, DF1312, and DB1503) were characterized as BVDV–1m. No BVDV–2, BVDV–3, or BDV was detected in field isolates by phylogenetic analysis.

## 4. Discussion

In this study, we identified 12 selected BVDV-1-positive samples that represented different flocks or geographical origins of blood samples collected between 2013 and 2016. Experimental or natural infection in goats has been previously confirmed [19]. Goats infected with BVDV represent a great risk for disease transmission, thus making the identification of BVDV infection in goats an important endeavor. This investigation described the genetic diversity of BVDV isolated from goats in southwestern China and demonstrated the occurrence of at least five subtypes of BVDV, including BVDV–1a, 1b, 1c, 1m, and 1p.

**Figure 1:**
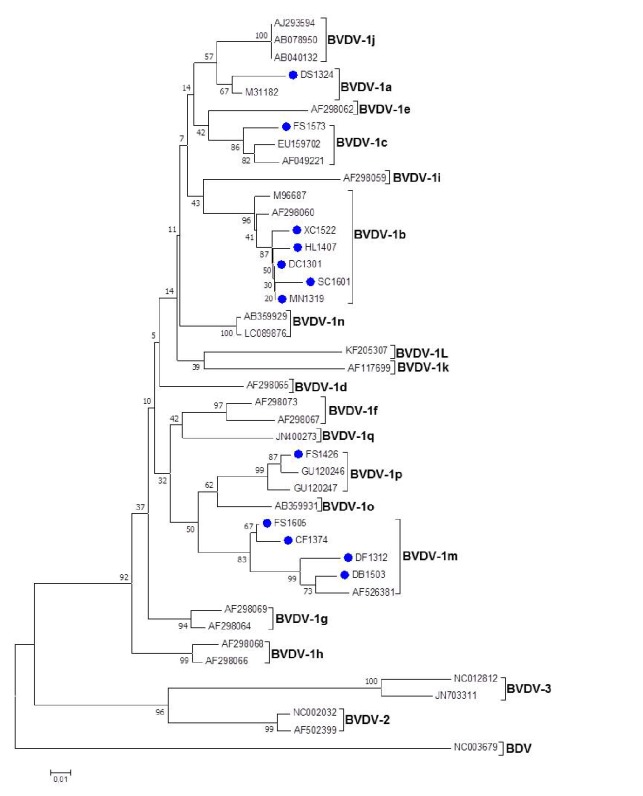
Phylogenetic tree of a 224 bp portion of the 5′-UTR nucleotide sequences. The BVDV strains isolated from goats in southwestern China, with Pestivirus reference strains of BVDV-1, BVDV-2, BVDV-3, and BDV registered in GenBank database. The tree was constructed using the Neighbor-Joining method, bootstrapping (1,000 replications), and the Kimura three parameter statistical model of MEGA 7.0 software. The 12 isolated sequences from goat serum in this study are designated with “blue circle”.

Among the BVDV–1 types, the most frequent subtype was BVDV–1b (n=5) (Table 1 and Figure 1). Five BVDV–1b-positive samples from different counties of southwestern China shared 98.1%–99.0% identity with each other and had 91.6%–98.0% identity with the reference strain, BVDV–1b 24–15 (AF298060), and Osloss (M96687). Those five strains were also clustered with the two BVDV-1b reference strains (Figure 1). In cattle, BVDV–1b was first detected in the tissue of a bovine fetus in 1980 in China and continually isolated and identified in Tianjin, Hebei, Gansu, Xinjiang, and Heilongjiang province from then onward [8, 20, 21].

The 1b subtype is widely considered to be a major contributor to infection in Chinese cattle [20, 22]. Similarly, BVDV-1b is also a main subtype in Chinese goat herds [13]. BVDV transmission among different animal species has been proven and, therefore, increased surveillance of BVDV infection in goats should be an important factor to consider in the control and understanding of BVDV infection in cattle.

BVDV–1m is another major subtype in this region (Figure 1). Four BVDV-1-positive samples identified in this study were classified as BVDV–1m. The isolates, FS1605, CF1374, DF1312, and DB1503, had 94.5%–98.7% homology with each other and shared 95.4%–97.0% homology with the BVDV–1m reference strain ZM–95 (AF526381) (Table 1). Therefore, those four positive samples were clustered into BVDV–1m. BVDV–1m was first isolated from a diseased pig in 1995 in China [23]. According to a survey conducted between 2005 and 2013, this subtype has been widely distributed in China and is the most prevalent among BVDV–1 subtypes [12].

Our positive samples, DS1324, FS1573, and FS1426, respectively, shared 87.9%, 97.5%, and 98.4% sequence identity with the reference strains BVDV–1a NADL (M31182), BVDV–1c Manasi (EU159702), Bega (AF049221), BVDV–1p TJ06 (GU120246), and TJ07 (GU120247) (Table 1). These positive samples were classified into BVDV–1a, 1c, and 1p, which are not commonly found.

In summary, this study demonstrated that BVDV–1 was a common causative agent for infection of goats in southwestern China and that 17.51% of the detected samples from ill goats were BVDV–1 positive. However, we did not assess samples from the healthy goats and cannot determine the prevalence of BVDV in healthy goats. To further our understanding of the genetic diversity of BVDV strains detected, all antigen-positive samples were confirmed and characterized with RT-PCR as being infected by BVDV–1, but not BVDV–2, BVDV–3, or BDV. Twelve BVDV–1-positive samples were identified from these RT-PCR positive samples and were clustered into the six subtypes BVDV–1a, 1b, 1c, 1m, and 1p. Due to the possibility of cross infections of BVDV that might occur among cattle, piglets, and goats, much more emphasis should be placed on epidemiology surveys in these animals. This is the first description of the genetic diversity of BVDV–1-positive samples collected from goats in southwestern China.

## Figures and Tables

**Table 1:** Description of the 12 isolates used in this study.

Name of the positive samples	Year	Symptoms	Region of the samples collected	GenBank Accession number	subtype	Identity (%)				
						1a NADL	1b Osloss	1c Mannasi	1m ZM95	1p TJ06
DF1312	2013	Respiratory symptom	DF	MG323517	1m				95.4	
DC1301	2013	Diarrhea	DC	MG323518	1b		98.0			
DS1324	2013	Weak	DS	MG323520	1a	87.9				
MN1319	2013	Diarrhea	MN	MG323522	1b		98.0			
CF1374	2013	Diarrhea	CF	MG323527	1m				95.7	
HL1407	2014	Abortion	HL	MG323523	1b		91.6			
FS1426	2014	Weak	FS	MG323525	1p					98.4
XC1522	2015	Mucosal disease	XC	MG323516	1b		97.0			
DB1503	2015	Abortion	DB	MG323519	1m				97.0	
FS1573	2015	Respiratory symptom	FS	MG323524	1c			97.5		
FS1605	2016	Respiratory symptom	FS	MG323521	1m				95.7	
SC1601	2016	Respiratory symptom	SC	MG323526	1b		97.0			

## Data Availability

The data used to support the findings of this study are included within the article.
